# Supplementary material for: From sequence to enzyme mechanism using multi-label machine learning
Source: BMC Bioinformatics. 2014 May 19;15:150. doi: 10.1186/1471-2105-15-150 (PMC4229970; doi:10.1186/1471-2105-15-150)
Supplement: Additional file 2 — Java code of ml2db. Additional file ml2db_code.tar.gz contains the Java source code to run the multi-label machine learning experiments and save the results to database. The code’s Javadoc is included. [file 1471-2105-15-150-S2.zip › additional file 2/ml2db/ml2db/doc/main/ml2db/al/utility/confidence/ConfidenceMetric.html]

ConfidenceMetric


JavaScript is disabled on your browser.


- Overview
- Package
- Class
- Use
- Tree
- Deprecated
- Index
- Help

- Prev Class
- Next Class

- Frames
- No Frames

- All Classes

- Summary:
- Nested |
- Field |
- Constr |
- Method

- Detail:
- Field |
- Constr |
- Method


main.ml2db.al.utility.confidence

## Class ConfidenceMetric

- java.lang.Object
- - main.ml2db.al.utility.UtilityMetric
  - - main.ml2db.al.utility.confidence.ConfidenceMetric

- ---

    

  ```
  public class ConfidenceMetric
  extends UtilityMetric
  ```

  Combines a Positioning metric (a metric that considers positively if an
  instance is close to other unlabelled instances and distant from other
  labelled instances) with the average certainty of the instance prediction
  (that is, how distant the average of confidence over all labels is from 50%)

  Version:
  :   7 Jul 2011

  Author:
  :   Luna De Ferrari ldf(at)st-andrews.ac.uk

- - ### Field Summary

    Fields

    | Modifier and Type | Field and Description |
    | --- | --- |
    | `static boolean` | `m_debug` |
    | `static java.lang.String` | `NAME` |
  - ### Constructor Summary

    Constructors

    Constructor and Description

    `ConfidenceMetric()`
  - ### Method Summary

    Methods

    | Modifier and Type | Method and Description |
    | --- | --- |
    | `ConfidenceCalculator` | `getConfidenceCalculator()` |
    | `java.lang.String` | `getMetricName()` |
    | `double` | `getUtility(mulan.classifier.MultiLabelLearner trainedLearner, IdInstance instance)` Gets a measure of how distant the instance is from other unlabelled instances (good if it's close to them) and labelled instances (bad if it's close to them) and multiplies it by the average confidence certainty (see Confidence class). |
    | `void` | `initialise()` |
    | `void` | `refresh(IdMultiLabelInstances labelled, IdMultiLabelInstances unlabelled, IdInstance queryInstance)` Refresh the set of labelled and unlabelled instances |

    - ### Methods inherited from class main.ml2db.al.utility.UtilityMetric

      `getUnlabelled, setUnlabelled`
    - ### Methods inherited from class java.lang.Object

      `equals, getClass, hashCode, notify, notifyAll, toString, wait, wait, wait`

- - ### Field Detail


    - #### NAME

      ```
      public static final java.lang.String NAME
      ```

      See Also:
      :   Constant Field Values


    - #### m\_debug

      ```
      public static final boolean m_debug
      ```

      See Also:
      :   Constant Field Values
  - ### Constructor Detail


    - #### ConfidenceMetric

      ```
      public ConfidenceMetric()
      ```
  - ### Method Detail


    - #### getConfidenceCalculator

      ```
      public ConfidenceCalculator getConfidenceCalculator()
      ```


    - #### getMetricName

      ```
      public java.lang.String getMetricName()
      ```

      **Specified by:**
      :   `getMetricName` in class `UtilityMetric`


    - #### getUtility

      ```
      public double getUtility(mulan.classifier.MultiLabelLearner trainedLearner,
                      IdInstance instance)
      ```

      Gets a measure of how distant the instance is from other unlabelled
      instances (good if it's close to them) and labelled instances (bad if
      it's close to them) and multiplies it by the average confidence certainty
      (see Confidence class).

      **Specified by:**
      :   `getUtility` in class `UtilityMetric`

      Parameters:
      :   `balance` - if > 0.5, the distance from labelled instances counts more
          than the closeness to many unlabelled instances. The other way
          round if < 0.5.
      :   `distancesFromLabelled` - the distances between this instance and each labelled instance
      :   `distancesFromUnlabelled` - the distances between this instance and each unlabelled
          instance

      Returns:
      :   a value representing the position of this instance in relation to
          other labelled and unlabelled instances.


    - #### initialise

      ```
      public void initialise()
      ```

      **Specified by:**
      :   `initialise` in class `UtilityMetric`


    - #### refresh

      ```
      public void refresh(IdMultiLabelInstances labelled,
                 IdMultiLabelInstances unlabelled,
                 IdInstance queryInstance)
      ```

      **Description copied from class: `UtilityMetric`**

      Refresh the set of labelled and unlabelled instances

      **Specified by:**
      :   `refresh` in class `UtilityMetric`


- Overview
- Package
- Class
- Use
- Tree
- Deprecated
- Index
- Help

- Prev Class
- Next Class

- Frames
- No Frames

- All Classes

- Summary:
- Nested |
- Field |
- Constr |
- Method

- Detail:
- Field |
- Constr |
- Method
